# Commentary: Predicting adverse outcomes in pregnant patients positive for SARS-CoV-2 by a machine learning approach

**DOI:** 10.1186/s12884-023-05864-3

**Published:** 2023-08-02

**Authors:** Noemi Salmeri, Massimo Candiani, Paolo Ivo Cavoretto

**Affiliations:** 1 Gynecology and Obstetrics Unit, IRCCS San Raffaele Scientific Institute, Milan, 20132 Italy; 2 Vita-Salute San Raffaele University, Milan, 20132 Italy

**Keywords:** SARS-CoV-2, COVID-19, Pregnancy, Machine learning, Artificial intelligence

## Abstract

SARS-CoV-2 infection poses a significant risk increase for adverse pregnancy outcomes both from maternal and fetal sides. A recent publication in *BMC Pregnancy and Childbirth* presented a machine learning algorithm to predict this risk. This commentary will discuss potential implications and applications of this study for future global health policies.

## Background

SARS-Cov-2 (Severe Acute Respiratory Coronavirus 2) infection confers a significant risk increase for adverse pregnancy outcomes, including maternal-neonatal morbidity and mortality, pre-eclampsia, maternal adiposity and gestational diabetes, preterm birth, stillbirth, and perinatal death [1–6]. The risk is higher when the infection occurs during the first or second trimester of pregnancy, necessitating increased monitoring and prenatal care [6]. Despite a growing body of evidence, no study has used artificial intelligence (AI), machine learning (ML), or data mining to inform global healthcare policies for COVID-19 in pregnancy. A recent publication in *BMC Pregnancy and Childbirth* by Young and collaborators [7] filled this knowledge gap with a retrospective cohort study of 1501 pregnancies testing positive for SARS-CoV-2-infection. The authors used ML models aiming at prediction of adverse pregnancy outcomes, stratified as those affecting the mother, fetus or the delivery. The ML algorithm was trained on retrospective international dataset, gathered from three countries (Canada, Austria, and Germany) and employed a set of 25 selected variables including demographics, patient and pregnancy characteristics (BMI, gravidity, parity, pre-existing diseases, maternal age, smoking, medications, multiple pregnancy, fetal growth, etc.), patient comorbidities, respiratory parameters, presenting symptoms, clinical signs and laboratory markers collected at timing of positive test (e.g., Soluble fms-like tyrosine kinase 1 and placental growth factor ratio, thrombocytopenia, haemoglobin, transaminase or haptoglobin levels, etc.). The model achieved a remarkable and clinically relevant accuracy of about 90%.

### Cautions in addressing prediction of adverse obstetric outcomes related to Covid-19

Although several studies developed COVID-19 prognostic models using ML, that of Young et al. is the first to present a specific algorithm for the particularly vulnerable population of pregnant individuals. Despite the model needing external validation before it can be recommended for routine clinical use, it potentially presents clinically relevant results, favoring the identification of individuals at high-risk that can benefit of additional surveillance, early hospital admission and therapy.

The risks of adverse pregnancy outcomes associated with SARS-CoV-2 infection are well established, with placental inflammation identified as a primary pathogenetic mechanism encompassing a cascade of events eventually leading to placental dysfunction [8]. However, most of the currently available knowledge was collected during earlier waves of the pandemic, when the predominant SARS-CoV-2 variants were different from those currently present. Since the introduction of safe and effective COVID-19 vaccines, a great step was achieved toward prevention of maternal-neonatal morbidity and mortality due to COVID-19 in pregnancy with significant lessening of the clinical course and reduced risks of obstetric complications with the additional benefit of conferring passive immunity the neonates. Recent major evidence showed that COVID-19 vaccination during pregnancy is safe and effective [9], even against the latest emerging viral variants, including Omicron, with significantly reduced rates of complications in vaccinated individuals [10].

In view of this, it is unfortunate that Young and colleagues did not include vaccination status and viral variants of concern in their prognostic model, however we believe these can be easily added in the future. Viruses have a highly variable and mutagenic nature, and global health policies use various strategies during pandemic waves, including vaccinations. These factors may render a single model inadequate to account for all this variability. Finally, the current model explores a composite outcome group, which does not allow specific indications on the type of interventions to be applied.

As such, the model proposed by Young and colleagues showed the limitations described, however they do not hamper its clinical utility and potential for being updated prospectively.

### Implications and future directions of machine learning approaches in Covid-19 era

COVID-19 pandemic broke out during the era of big data and exponential growth in data analytics.

Indeed, in the past two decades, the applications of AI and ML in healthcare have experienced significant advancements due to the maturity of electronic health records and the introduction of ML-based clinical decision support systems. These technologies have been widely implemented in healthcare to address Covid-19-related challenges, supporting decision-making and providing enormous social value in safeguarding public health. Bastani and colleagues [11] proposed an efficient and targeted COVID-19 border testing approach using a reinforcement learning system named Eva. Similarly, Pfaff et al. [12] developed an Extreme Gradient Boosting (XGboost) ML model capable of accurately identifying potential long COVID-19 patients, with high efficiency and accuracy.

The study by Young and colleagues introduced a high-performing ML-based prognostic tool specifically for pregnant individuals with COVID-19. The authors tested several ML models and found that the Random Forest model achieved the best performance, correctly identifying the outcome in 83.3% of high-risk patients and 92.5% of low-risk patients, with an overall accuracy of 89.0%, an AUC of 0.90 (95% CI 0.83 to 0.95), and recall, precision, and F1 score of 0.85, 0.94, and 0.89, respectively. The algorithm was implemented using a Random Forest model, an ensemble learning method that constructs multiple decision trees during training and outputs the mean prediction (regression) of the individual trees [13]. The multiple decision trees are built on a random subset of features and data points, which improve the model’s generalization and reduce the likelihood of overfitting [13]. The Random Forest model as compared to simpler models (such as single decision trees) offers the advantage of estimating features importance, assisting in their selection. Data mining is essential for extracting valuable information from large volumes of data and transforming it into an interpretable structure for further analysis. This is particularly important for COVID-19, as the infection has been highly variable over time due to the influence of different virus variants and mutable population immunity.

## Conclusions

Despite some limitations, the model presented by Young and collaborators can help identifying early warning signs by an AI and ML workflow. This will facilitate a shift towards personalized medicine, with patient-based monitoring and care tailored to their expected prognosis. The need to shift healthcare delivery from a passive approach to a proactive and predictive method is becoming crucial and powerful ML-based models of outcome prediction are required in current medical practice to achieve this aim. Despite COVID-19 “no longer constitutes a public health emergency of international concern” as declared in a very recent World Health Organization statement [14], SARS-Cov-2 did not vanish, and we must continue supporting research and appropriate measures minimizing global health impact.

The prediction model object of this editorial showed clinical usefulness for identifying individuals at greater risk of adverse pregnancy outcomes during COVID-19 infections, and showed the potential for translation to other similar clinical conditions (future epidemics due to respiratory viruses), and to specific single outcomes (rather than to composite measure of abnormal outcome), assuming model external validation, adaptability and updating to changing circumstances. Based on this knowledge, future research may transfer this experience into novel projects creating robust and reliable models utilizing AI and ML approaches to be used in global health policy management of comparable forthcoming circumstances.

## Data Availability

Not applicable.

## Abbreviations

SARS-Cov-2: Severe Acute Respiratory Coronavirus 2
COVID-19: Coronavirus disease
ML: machine learning
AI: artificial intelligence
AUC: area under curve
WHO: World Health Organization
